# Behavioral Evidence for a Tau and HIV-gp120 Interaction

**DOI:** 10.3390/ijms23105514

**Published:** 2022-05-15

**Authors:** Murali Vijayan, Linda Yin, P. Hemachandra Reddy, Khalid Benamar

**Affiliations:** 1 Internal Medicine Department, Texas Tech University Health Sciences Center, 3601 4th Street, Lubbock, TX 79430, USA; murali.vijayan@ttuhsc.edu (M.V.); hemachandra.reddy@ttuhsc.edu (P.H.R.); 2 Garrison Institute on Aging, Texas Tech University Health Sciences Center, Lubbock, TX 79430, USA; linda.yin@ttuhsc.edu; 3 Department of Pharmacology and Neuroscience, School of Medicine Lubbock, Texas Tech University Health Sciences Center, Lubbock, TX 79430, USA

**Keywords:** human immunodeficiency virus-1 (HIV), combination antiretroviral therapy (cART), HIV-associated neurocognitive disorder (HAND), intracerebroventricularly (ICV), Alzheimer’s disease (AD), central nervous system (CNS), cerebrospinal fluid (CSF), glycoprotein 120 (gp-120)

## Abstract

Despite successful virologic control with combination antiretroviral therapy (cART), about half of people living with the human immunodeficiency virus-1 (HIV) develop an HIV-associated neurocognitive disorder (HAND). It is estimated that 50% of individuals who are HIV-positive in the United States are aged 50 years or older. Therefore, a new challenge looms as individuals living with HIV increase in age. There is concern that Alzheimer’s disease (AD) may become prevalent with an earlier onset of cognitive decline in people living with HIV (PLWH). Clinical data studies reported the presence of AD biomarkers in PLWH. However, the functional significance of the interaction between HIV or HIV viral proteins and AD biomarkers is still not well studied. The main goal of the present study is to address this knowledge gap by determining if the HIV envelope glycoprotein 120 (HIV-gp120) can affect the cognitive functions in the Tau mouse AD model. Male Tau and age-matched, wild-type (WT) control mice were treated intracerebroventricularly (ICV) with HIV-gp120. The animals were evaluated for cognitive function using a Y-maze. We found that HIV-gp120 altered cognitive function in Tau mice. Notably, HIV-gp120 was able to promote a cognitive decline in transgenic Tau (P301L) mice compared to the control (HIV-gp120 and WT). We provide the first in vivo evidence of a cognitive interaction between an HIV viral protein and Tau mice.

## 1. Introduction

Alzheimer’s disease (AD) is a devastating neurodegenerative disorder and one of the most common causes of neurodegenerative dementia in the elderly. It is characterized by a progressive impairment of cognitive functions. Currently, over 50 million people worldwide live with AD-related dementia, and this number is expected to increase to 131.5 million by 2050 [1,2]. The disease is neuropathologically characterized by the deposition of abnormal proteins, resulting in the formation of extracellular senile plaques and intracellular neurofibrillary tangles (NFTs). The senile plaques contain primarily neurotoxic amyloid-β (Aβ) whereas NFTs consist of abnormal hyperphosphorylated Tau aggregates. Phosphorylated Tau disrupts the microtubule assembly, leading to defective axonal transport, ultimately leading to synaptic starvation and neuronal death.

In 2018, an estimated 37.9 million people were living with human immunodeficiency virus-1 (HIV) [3,4]. HIV-associated neurocognitive disorder (HAND) is a common primary neurological disorder of the central nervous system, associated with an HIV infection. Despite successful virologic control with combination antiretroviral therapy (cART), about half of people living with HIV (PLWH) develop HAND. It is estimated that 50% of the U.S. HIV-positive population is aged 50 years or older. Therefore, a new challenge looms as individuals living with an HIV infection age. There is concern that AD may become prevalent, with an earlier onset of cognitive decline in PLWH.

Clinical data derived mostly from case studies reported the presence of AD biomarkers in PLWH [5,6,7]. For example, a virally-suppressed female case (52 years old) demonstrated AD neuropathology and age-related Tau astrogliopathy in the hippocampus [5]. Consistent with AD, the 52-year-old demonstrated severe learning, memory, and daily function impairments [5]. AD biomarkers (e.g., Aβ_1–42,_ Tau) are found in the cerebrospinal fluid (CSF) of HAND patients [8].

Preclinical data support a potential interaction between HIV viral proteins, such as HIV envelope glycoprotein 120 (HIV-gp120), and AD biomarkers. For example, increased Tau phosphorylation has been reported in HIV-gp120 transgenic mice [9]. The treatment of primary hippocampal cell cultures with recombinant HIV-gp120 promoted Aβ_1–42_ secretion [10]. Aβ deposition was accelerated in HIV-gp120/APP/PS1 mice compared with APP/PS1 mice in the dentate gyrus [11,12,13,14,15].

Despite this evidence, the functional significance of HIV or HIV viral proteins and AD interaction is still poorly understood. The main goal of the present study is to address this knowledge gap by determining if HIV-gp120 can affect the cognitive function in the transgenic Tau mouse model.

## 2. Results

### 2.1. HIV-gp120 Promotes the Onset of Cognitive Deficit of Tau Mice

**Figure 1:**
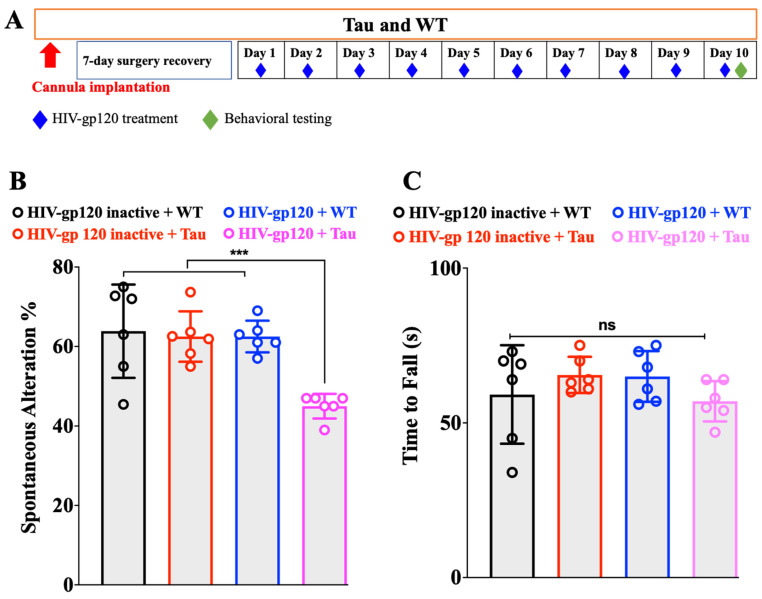
(**A**) Experimental design. (**B**) HIV-gp120 promoted the onset of cognitive deficit of Tau mice. Effects of HIV-gp120 on motor coordination (**C**). *** *p* < 0.001, ns: not significant. Data are presented as mean ± SEM.

We used 4-month-old, an age prior to the onset of cognitive deficit, male Tau mice [16]. The HIV-gp120 dose we used does not produce cognitive decline by itself. Therefore, any effect on cognition during this testing period was the result of HIV-gp120 and Tau interaction and not their individual effects. The design of the experiment is shown schematically in Figure 1A.

Male Tau and age-matched WT mice were treated ICV with HIV-gp120 IIIB (1 ng/2 µL) or the control (heat-inactivated gp120 (90 °C for 30 min), 1 ng/2 µL) daily for 10 days. The animals were evaluated in the Y-maze as we previously reported [17]. In comparison with the control (inactivated HIV-gp120), HIV-gp120 (at a dose that has no effect on the Y-maze) treatment induced a significant Y-maze spontaneous alternation deficit (Figure 1B, one-way ANOVA, *p* < 0.001, number of mice, n = 6) in Tau mice. WT treated with HIV-gp120 or inactivated HIV-gp120 had no effect on the cognitive function (Figure 1B, one-way ANOVA, *p* > 0.05, n = 6). The fact that the control group (inactivated HIV-gp120 + Tau) did not show any effect on the Y-maze test demonstrated that during the treatment time (10 days), the Tau mice did not develop cognitive deficits (independent of HIV-gp120 action) that could be confounded with HIV-gp120 action. 

For enhanced rigor, we also monitored the mice for motor impairment that could impact performance in the Y-maze and confound the assessment of cognition. A rotarod apparatus (Panlab, Harvard Apparatus, Holliston, MA, USA) was used. Tau mice showed a similar performance on the accelerating rotarod compared to same-aged WT (Figure 1C, one-way ANOVA, *p* > 0.05, n = 6). 

### 2.2. Effect of Mitochondrial Division Inhibitor 1 (Mdivi1) on the Cognitive Interaction between HIV-gp120 and Tau Mice

**Figure 2:**
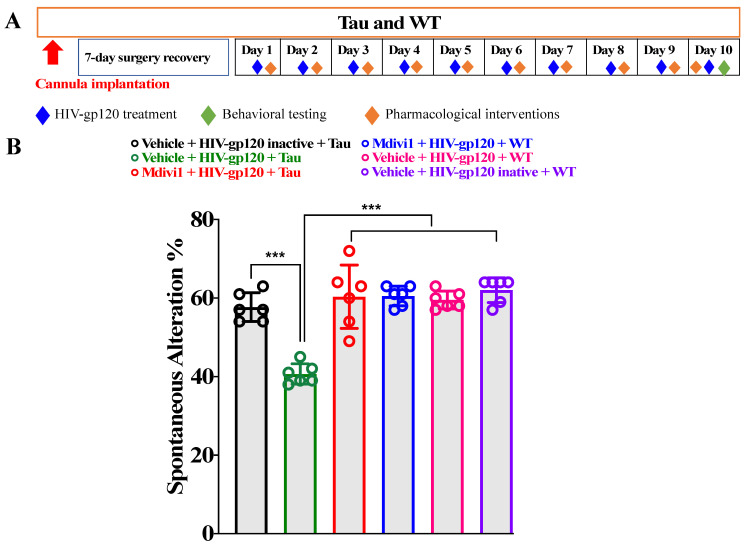
(**A**) Experimental design. (**B**) Mdivi1 prevented the cognitive interaction between HIV-gp120 and Tau mice. *** *p* < 0.001. Data are presented as mean ± SEM.

The design of the experiment is shown schematically in Figure 2A. Compared to the vehicle (dimethylsulphoxide + saline), the pretreatment with mitochondrial division inhibitor 1 (Mdivi1, 20 mg/kg intraperitoneal (i.p.) attenuated the effect of HIV-gp120 in Tau mice in the Y-maze test (Figure 2B, one-way ANOVA, *p* < 0.001, n = 6). The Y-maze test was conducted starting 1 hour post Mdivi1 injection [18]. The dose and treatment time were based on the pharmacokinetics of Mdivi1 [18].

## 3. Discussion

In light of our research, we are reporting two key findings. First, HIV-gp120 alters the cognitive function of Tau mice. Second, Mdivi1 prevents the cognitive interaction between HIV-gp120 and Tau mice.

The brain is one of the first sites targeted by HIV. This virus enters the brain early in the disease process and then continues to produce central nervous system (CNS) dysfunction as the disease progresses. HIV enters target cells by binding its HIV envelope HIV-gp120. This viral protein produces several neurobehavioral effects in rodents that are characteristic of HIV/AIDS [19,20,21,22]. We used the Tau AD mouse model to determine any behavioral interaction with HIV-gp120. Tau transgenic mice were generated with a human Tau P301L mutation [23]. Signs of cognitive impairments were detected at 5 months [16]. Recently, we found these effects became more dramatically impaired as the mice aged [24]. Here, we injected HIV-gp120 directly into the brain and determined its effect on cognitive function in Tau mice. We found that HIV-gp120 altered cognitive function in Tau mice. Notably, HIV-gp120 was able to promote a cognitive decline in Tau mice compared to the control (HIV-gp120 and WT). 

A role of abnormal mitochondrial dynamics has been found to be an early event in the AD disease process in studies using postmortem AD brains, AD cell cultures, and AD mouse models [25,26,27,28,29,30,31,32]. HIV altered neuronal mitochondrial fission/fusion although different patterns have been reported [33,34,35]. Exposure of human primary neurons to HIV-gp120 accelerates the balance of mitochondrial dynamics toward fission (fragmented mitochondria) and induces perinuclear aggregation of mitochondria and mitochondrial translocation of dynamin-related protein 1 (DRP1), leading to neuronal mitochondrial fragmentation [35]. In vitro, recombinant HIV-gp120 decreased the total and active dynamin 1-like levels in primary neurons [33]. Our data show that the pretreatment with a mitochondrial division inhibitor prevents the cognitive interaction between HIV-gp120 and Tau mice. Based on this background and our pharmacological data, it seems as though the HIV-gp120 promotes the onset of cognitive decline in Tau mice through a mechanism that involves the enhancement of mitochondrial fragmentation, and this deficit can be rescued with Mdivi-1. The focus on mitochondria does not negate the role of other mechanisms. For example, clinical [1,2,3,4] and preclinical [5] data have shown neuroinflammation in the brains of people with AD as well as in people living with HIV [6]. In AD, neuroinflammation, instead of being a mere bystander activated by emerging senile plaques and neurofibrillary tangles, contributes as much or more to the pathogenesis as do the plaques and tangles themselves. Tau can induce inflammation [7] and studies show that microglial activation contributes to Tau pathology during AD pathogenesis [8,9,10,11]. HIV-gp120 can also produce neuroinflammation [12]. Therefore, a synergistic effect on neuroinflammation may account for HIV-gp120, promoting cognitive decline in Tau mice.

In summary, the current studies provide in vivo evidence of a cognitive interaction between an HIV viral protein and an AD mouse model. These data suggest that HIV and AD are not independent conditions, but they can interact with each other when they are comorbid. These data suggest a contribution of mitochondria in the cognitive interaction between HIV-gp120 and Tau mice. 

## 4. Methods

### 4.1. Animals

Experiments were performed using male transgenic Tau (P301L mutation) mice (4 months old) from Taconic Farms (Cambridge City, IN, USA). Mice used in these experiments were housed in groups (four per cage) under a 12:12 h light−dark cycle (lights on at 07:00 and lights off at 19:00) and provided with standard mouse chow *ad libitum*. All animal care and experimental procedures used in this study were approved by the Institutional Animal Care and Use Committee (IACUC) of the Texas Tech University Health Sciences Center and conducted in accordance with the National Institutes of Health’s accepted guidelines found in the Guide for the Care and Use of Laboratory Animals.

### 4.2. Behavior

The Y-maze was used to quantify cognitive deficits and performed as described earlier [17]. The Y-maze was performed with video tracking software (Ethovision) using an overhead video camera system to automate behavioral testing. We first placed a mouse in a randomly selected start arm of the Y-maze. Upon leaving the start arm, the mouse chose between entering either the left or the right goal arm. With repeated trials, a mouse with no cognitive impairment typically showed less of a tendency to enter a previously visited arm. To determine the rates of spontaneous alternation, each mouse was allowed to explore the Y-maze for 5 min. Spontaneous alternation was defined as successive entries into three different passages (A, B, and C) without repetition (e.g., ABC or BCA but not ABA). 

Motor impairment was tested using the accelerating rotarod test (Model LE8205, Harvard Apparatus, Holliston, MA, USA). Mice were placed on a rotating rod revolving at 4 RPM, which was programmed to accelerate to 40 RPM over the course of 5 min. The latency to fall from the rotarod was recorded in seconds. Mice were trained for 3 days on the rotarod before experimental testing began. 

### 4.3. Cannula Implantation

Briefly, for cannula implantation (for intracerebroventricularly (ICV) administrations, mice were anesthetized with isoflurane (4% induction and 2% maintenance) [36,37]. The animals were placed in a digital stereotaxic alignment system (David Kopf Instruments, Model 942), and a small incision was made on the skull in addition to a tiny hole to allow the insertion of sterile guide cannulas. A sterilized stainless steel guide cannula (33-gauge (C3151), Plastics One Inc., Roanoke, VA, USA) was implanted (the stereotaxic coordinates are as follows: 0.46 mm posterior to the bregma, 1 mm from the midline, and 2 mm down from the surface of the skull). [36,37] Mice were assessed for health and sickness-type behavior (e.g., change in body temperature and decrease in body weight) routinely after surgery. After a 7-day recovery period, HIV-gp120 or inactivated HIV-gp120 was microinjected ICV via an internal cannula (Plastics One Inc., Roanoke, VA, USA) in awake mice. The internal cannula (Plastics One Inc., Roanoke, VA, USA) was left in place for an additional 90 s to allow diffusion. Immediately thereafter, a dummy cannula was inserted into the cannula guide to prevent contamination. 

### 4.4. Statistical and Histologic Analysis

Statistics were performed using Graph Pad Prism 9 (GraphPad Software, San Diego, CA, USA). Spontaneous alteration and motor performance data were analyzed using one-way analysis of variance (ANOVA) with the Bonferroni post hoc test. A *p* value of <0.05 was considered significant. At the end of the behavioral experiments, standard histological procedures were used to verify the site of injection [36,37,38,39].

### 4.5. Drugs

HIV-gp120 IIIB was purchased from Biological Life Science (catalog # H6003-34E). The mitochondrial division inhibitor 1 (Mdivi-1) (catalog # 338967-87-6), dimethylsulphoxide, and saline were purchased from Sigma.

## Data Availability

Not applicable.

## References

[1] (2013). The Global Impact of Dementia 2013–2050. https://www.alz.co.uk/research/GlobalImpactDementia2013.pdf.

[2] Wimo A., Guerchet M., Ali G.C., Wu Y.T., Prina A.M., Winblad B., Jonsson L., Liu Z., Prince M. (2017). The worldwide costs of dementia 2015 and comparisons with 2010. Alzheimers Dement..

[3] Global HIV & AIDS Statistics 2019. https://www.unaids.org/en/resources/fact-sheet.

[4] (2016). Diagnoses of HIV Infection in the United States and Dependent Areas. https://www.cdc.gov/hiv/pdf/library/reports/surveillance/cdc-hiv-surveillance-report-2016-vol-28.pdf.

[5] Morgello S., Jacobs M., Murray J., Byrd D., Neibart E., Mintz L., Meloni G., Chon C., Crary J. (2018). Alzheimer’s disease neuropathology may not predict functional impairment in HIV: A report of two individuals. J. Neurovirol..

[6] Tripathi M., Yadav S., Kumar V., Kumar R., Tripathi M., Gaikwad S., Kumar P., Bal C. (2016). HIV encephalitis with subcortical tau deposition: Imaging pathology in vivo using F-18 THK 5117. Eur. J. Nucl. Med. Mol. Imag..

[7] Turner R.S., Chadwick M., Horton W.A., Simon G.L., Jiang X., Esposito G. (2016). An individual with human immunodeficiency virus, dementia, and central nervous system amyloid deposition. Alzheimers Dement..

[8] Clifford D.B., Fagan A.M., Holtzman D.M., Morris J.C., Teshome M., Shah A.R., Kauwe J.S. (2009). CSF biomarkers of Alzheimer disease in HIV-associated neurologic disease. Neurology.

[9] Patrick C., Crews L., Desplats P., Dumaop W., Rockenstein E., Achim C.L., Everall I.P., Masliah E. (2011). Increased CDK5 expression in HIV encephalitis contributes to neurodegeneration via tau phosphorylation and is reversed with Roscovitine. Am. J. Pathol..

[10] Aksenov M.Y., Aksenova M.V., Mactutus C.F., Booze R.M. (2010). HIV-1 protein-mediated amyloidogenesis in rat hippocampal cell cultures. Neurosci. Lett..

[11] Bae M., Patel N., Xu H., Lee M., Tominaga-Yamanaka K., Nath A., Geiger J., Gorospe M., Mattson M.P., Haughey N.J. (2014). Activation of TRPML1 clears intraneuronal Abeta in preclinical models of HIV infection. J. Neurosci..

[12] Guindon J., Blanton H., Brauman S., Donckels K., Narasimhan M., Benamar K. (2019). Sex Differences in a Rodent Model of HIV-1-Associated Neuropathic Pain. Int. J. Mol. Sci..

[13] Herzberg U., Sagen J. (2001). Peripheral nerve exposure to HIV viral envelope protein gp120 induces neuropathic pain and spinal gliosis. J. Neuroimmunol..

[14] Wallace V.C., Blackbeard J., Segerdahl A.R., Hasnie F., Pheby T., McMahon S.B., Rice A.S. (2007). Characterization of rodent models of HIV-gp120 and anti-retroviral-associated neuropathic pain. Brain.

[15] Yuan S.B., Shi Y., Chen J., Zhou X., Li G., Gelman B.B., Lisinicchia J.G., Carlton S.M., Ferguson M.R., Tan A. (2014). Gp120 in the pathogenesis of human immunodeficiency virus-associated pain. Ann. Neurol..

[16] Arendash G.W., Lewis J., Leighty R.E., McGowan E., Cracchiolo J.R., Hutton M., Garcia M.F. (2004). Multi-metric behavioral comparison of APPsw and P301L models for Alzheimer’s disease: Linkage of poorer cognitive performance to tau pathology in forebrain. Brain Res..

[17] Vijayan M., George M., Bunquin L.E., Bose C., Reddy P.H. (2022). Protective effects of a small-molecule inhibitor DDQ against tau-induced toxicities in a transgenic tau mouse model of Alzheimer’s disease. Hum. Mol. Genet..

[18] Rappold P.M., Cui M., Grima J.C., Fan R.Z., de Mesy-Bentley K.L., Chen L., Zhuang X., Bowers W.J., Tieu K. (2014). Drp1 inhibition attenuates neurotoxicity and dopamine release deficits in vivo. Nat. Commun..

[19] Benamar K., Addou S., Yondorf M., Geller E.B., Eisenstein T.K., Adler M.W. (2010). Intrahypothalamic injection of the HIV-1 envelope glycoprotein induces fever via interaction with the chemokine system. J. Pharmacol. Exp. Ther..

[20] Barak O., Goshen I., Ben-Hur T., Weidenfeld J., Taylor A.N., Yirmiya R. (2002). Involvement of brain cytokines in the neurobehavioral disturbances induced by HIV-1 glycoprotein120. Brain Res..

[21] Opp M.R., Rady P.L., Hughes T.K., Cadet P., Tyring S.K., Smith E.M. (1996). Human immunodeficiency virus envelope glycoprotein 120 alters sleep and induces cytokine mRNA expression in rats. Am. J. Physiol..

[22] Shanmugam S., Patel D., Guindon J., Reddy P.H., Narasimhan M., Benamar K. (2020). Gene expression of endocannabinoid system in HIV-1-related neuropathic pain model. Biochim. Biophys. Acta Mol. Basis Dis..

[23] Lewis J., McGowan E., Rockwood J., Melrose H., Nacharaju P., Van Slegtenhorst M., Gwinn-Hardy K., Paul Murphy M., Baker M., Yu X. (2000). Neurofibrillary tangles, amyotrophy and progressive motor disturbance in mice expressing mutant (P301L) tau protein. Nat. Genet..

[24] Kandimalla R., Manczak M., Yin X., Wang R., Reddy P.H. (2018). Hippocampal phosphorylated tau induced cognitive decline, dendritic spine loss and mitochondrial abnormalities in a mouse model of Alzheimer’s disease. Hum. Mol. Genet..

[25] Reddy P.H., McWeeney S. (2006). Mapping cellular transcriptosomes in autopsied Alzheimer’s disease subjects and relevant animal models. Neurobiol. Aging.

[26] Reddy P.H., Mani G., Park B.S., Jacques J., Murdoch G., Whetsell W., Kaye J., Manczak M. (2005). Differential loss of synaptic proteins in Alzheimer’s disease: Implications for synaptic dysfunction. J. Alzheimers Dis..

[27] Manczak M., Calkins M.J., Reddy P.H. (2011). Impaired mitochondrial dynamics and abnormal interaction of amyloid beta with mitochondrial protein Drp1 in neurons from patients with Alzheimer’s disease: Implications for neuronal damage. Hum. Mol. Genet..

[28] Manczak M., Reddy P.H. (2012). Abnormal interaction of VDAC1 with amyloid beta and phosphorylated tau causes mitochondrial dysfunction in Alzheimer’s disease. Hum. Mol. Genet..

[29] Kandimalla R., Manczak M., Fry D., Suneetha Y., Sesaki H., Reddy P.H. (2016). Reduced dynamin-related protein 1 protects against phosphorylated Tau-induced mitochondrial dysfunction and synaptic damage in Alzheimer’s disease. Hum. Mol. Genet..

[30] Manczak M., Kandimalla R., Fry D., Sesaki H., Reddy P.H. (2016). Protective effects of reduced dynamin-related protein 1 against amyloid beta-induced mitochondrial dysfunction and synaptic damage in Alzheimer’s disease. Hum. Mol. Genet..

[31] Manczak M., Kandimalla R., Yin X., Reddy P.H. (2018). Hippocampal mutant APP and amyloid beta-induced cognitive decline, dendritic spine loss, defective autophagy, mitophagy and mitochondrial abnormalities in a mouse model of Alzheimer’s disease. Hum. Mol. Genet..

[32] Reddy P.H., Yin X., Manczak M., Kumar S., Pradeepkiran J.A., Vijayan M., Reddy A.P. (2018). Mutant APP and amyloid beta-induced defective autophagy, mitophagy, mitochondrial structural and functional changes and synaptic damage in hippocampal neurons from Alzheimer’s disease. Hum. Mol. Genet..

[33] Fields J.A., Serger E., Campos S., Divakaruni A.S., Kim C., Smith K., Trejo M., Adame A., Spencer B., Rockenstein E. (2016). HIV alters neuronal mitochondrial fission/fusion in the brain during HIV-associated neurocognitive disorders. Neurobiol. Dis..

[34] Rozzi S.J., Avdoshina V., Fields J.A., Mocchetti I. (2018). Human immunodeficiency virus Tat impairs mitochondrial fission in neurons. Cell Death Discov..

[35] Teodorof-Diedrich C., Spector S.A. (2018). Human Immunodeficiency Virus Type 1 gp120 and Tat Induce Mitochondrial Fragmentation and Incomplete Mitophagy in Human Neurons. J. Virol..

[36] Benamar K., Geller E.B., Adler M.W. (2008). Elevated level of the proinflammatory chemokine, RANTES/CCL5, in the periaqueductal grey causes hyperalgesia in rats. Eur. J. Pharmacol..

[37] Paxinos G.A.F.K. (2001). The Mouse Brain in Streotaxic Coordinates.

[38] Benamar K., Rawls S.M., Geller E.B., Adler M.W. (2004). Intrahypothalamic injection of deltorphin-II alters body temperature in rats. Brain Res..

[39] Benamar K., McMenamin M., Geller E.B., Chung Y.G., Pintar J.E., Adler M.W. (2005). Unresponsiveness of mu-opioid receptor knockout mice to lipopolysaccharide-induced fever. Br. J. Pharmacol..

